# Self-medication among health workers in a tertiary institution in South-West Nigeria

**DOI:** 10.11604/pamj.2016.24.312.8146

**Published:** 2016-08-16

**Authors:** Oluwole Adeyemi Babatunde, Joseph Olusesan Fadare, Olujide John Ojo, Kabir Adekunle Durowade, Oladele Ademola Atoyebi, Paul Oladapo Ajayi, Temitope Olaniyan

**Affiliations:** 1 Department of Community Medicine, Federal Medical Center, Ido-Ekiti, Nigeria; 2 Department of Pharmacology, College of Medicine, Ekiti State University, Ado-Ekiti, Nigeria; 3 Department of Community Medicine, Unilorin Teaching Hospital, Ilorin, Nigeria; 4 Department of Community Medicine, Federal Medical Center, P.M.B 201, Ido-Ekiti, Nigeria

**Keywords:** Self-medication, adverse drug reactions, healthcare workers, hospital

## Abstract

**Introduction:**

Inappropriate self-medication results in wastage of resources, resistance to pathogen and generally entails serious health hazard. This study was undertaken to determine the knowledge, practice and reasons for practice of self-medication among health workers in a Nigerian tertiary institution.

**Methods:**

This was a cross-sectional descriptive study conducted among staff of Federal Medical Center Ido-Ekiti, Nigeria. Simple random sampling technique was used to select 305 respondents that were interviewed via a pretested semi-structured questionnaire. Analysis was done using SPSS version 15 and while chi-square test was used to test significance between variables, significant (p value set<0.05).

**Results:**

Among the 305 respondents interviewed, the age range was 18-52yrs with greater proportion being males (51.8%). Majority of respondents were aware of self-medication (94.8%), but only 47.2% had good knowledge of it. Reasons for practicing self-medication were financial problem (10.8%), mild sickness (10.8%), lack of time (13.4%), knowledge of diagnosis (5.6%), convenience (2.3%) and non-availability of doctors (3.0%). The drugs used by respondents without prescription included analgesics (38.2%), antibiotics (19.0%) anti-malaria drugs (13.3%), and others (29.4%). Conditions for which respondents self-medicated were body pains (14.9%), catarrh (14.9%), headache (14.3%), sore throat (11.5%), diarrhea (11.2%), fever (9.0%) and toothache (5.6%).

**Conclusion:**

The study demonstrates that the prevalence of self-medication is relatively high. There is need for health education on the implication and danger of self-medication. There is also need for government to pass and enforce law to restrict free access to drugs.

## Introduction

It is common for people to feel unwell and human beings have an inherent tendency to use herbs, portion and medications for treating themselves [1]. Everyday people throughout the world practice self- care and in many instances, they do so through self-medication which is now increasingly being considered as a component of self- care [1]. World Health Organization (WHO) defines self-care as what people do by themselves to keep their health, prevent and treat illness [2]. The concept of self-medication is the selection and use of medicines chosen by the patient for the treatment of an illness or the treatment of symptoms perceived by him/her. The term responsible self-medication implies that the patient treat his illness or symptom with medicine available without prescription which is safe and effective when used according to the established conditions [2]. In April 7, 2011, World Health Organization’s theme was “antimicrobial resistance and its global spread” and it focused on the need for government and stakeholders to implement the policies and practices needed to prevent and counter the emergence of highly resistant microorganisms [3]. The fourth of the six point policy package to combat the spread of antimicrobial resistance is to regulate and promote rational use of medicine [3]. Antimicrobial resistance is one pitfall of self-medication [4, 5] and so also is antimalarial resistance [6]. It has been found in several studies that inappropriate self-medication results in wastage of resources, resistance to pathogen and generally entails serious health hazard such as drug reaction, prolong suffering, drug dependence, missed diagnosis and delay in appropriate treatment [1, 5–7]. The other problem noticed were wrong choice of antibiotic, use of insufficient dosage and unnecessary therapy [8, 9].

Self-medication is a global problem [10]. The prevalence from studies carried out in Europe is 68% [10] while it is higher in African countries (40.7-81.8%) [8]. Studies conducted in Kuwait, India and Nepal showed prevalence rates of 92%, 31% and 59% [10, 11] respectively. In Sudan and Cameroun prevalence of 73.9% and 55.7% were observed respectively [6], while a study in Nigeria showed that 85% of participants practiced self-medication [7]. A study in Greece showed that the most common ailment for which self-medication was practiced were throat symptom and bronchitis while main medication sources were pharmacies and medication leftovers from previous prescriptions [12]. Another study in Uganda showed that fever, cough, abdominal pain accounted for most common ailment while drug sources included drug shops, private clinics, ordinary shops and government health centers. The place where these drugs are gotten also serves as source of knowledge about the drugs [13]. Drugs that are commonly used for self-medication includes anti-malaria, (55.3%), analgesic (43.9%), antibacterial (21.7%). In another study, modern drugs accounted for 93.6%, homeopathic drugs 4.6% and herbal medicines 2% [14]. Reasons given for the practice of self-medication includes lack of money, ignorance, long distance to healthcare facility, mild/minor illness, poor attitude of health workers (rude, corrupt, dirty) re- treatment of similar illness and lack of health personnel [1, 13]. Health workers may differ from general population because they are exposed to the knowledge about disease and drugs. It will be interesting to know how this will affect their self-medication habit. The study was undertaken to determine the knowledge, practice and reason for practice of self-medication among health workers in tertiary hospital. The study will help policymakers in controlling self-medication in Nigeria by helping them to take informed decisions.

## Methods

This study was conducted among healthcare workers in Federal Medical Centre, Ido-Ekiti, a tertiary healthcare facility in Ekiti State, a south-western Nigeria. It was conducted over a period of three months (February to April 2012). At the time of this study, there were 1390 health workers including 305 doctors, 348 nurses, 37 community health extension workers, 40 laboratory scientist/technicians, 28 pharmacist, 15 radiographers, 7 physiotherapists, 340 health attendants, 3 optometrists and 267 others including speech therapists, medical psychologists, dieticians, nutritionists and health record officers and administrative staff. This was a cross-sectional descriptive study amongst health workers in the hospital. The minimum sample size for this study was determined using the Fisher’s formula. A sample size of 297 was derived. Proportional allocation of the sample sizes was done to the ten groups of staff based on their population. Simple random sampling technique by balloting was used to select respondents in each group. Pretested, semi-structured, self-administered questionnaire was used to generate quantitative data. The questionnaire was pretested at University Teaching Hospital, Ado-Ekiti, Ekiti State. The questionnaire included items relating to demographic characteristic, knowledge, practice and reason for practicing self-medication. Knowledge of self-medication was scored and respondents who had knowledge score of 0-3 were regarded as poor knowledge while those with scores between 4 and 8 as good knowledge. The questionnaires were self-administered and data was analyzed via SPSS version 15. Chi-square test was used to determine statistical significance of observed differences in cross tabulated variables while predetermined level of significance was set at p-value of less than 0.05. Clients’ consent was obtained before the interview. The natures of the study, participation status, benefit of the study and confidentiality issue were made clear to the respondents before obtaining their consent. Ethical clearance was obtained from ethical committee of the hospital prior to carrying out this study.

## Results

**Figure 1:**
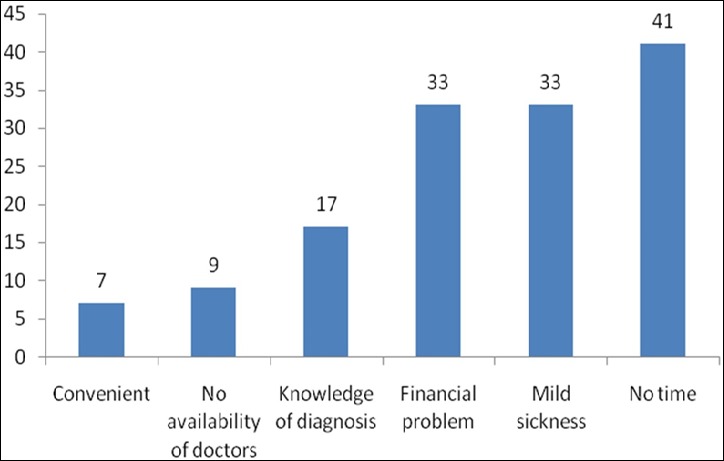
Reasons for self medication

The total number of people interviewed was 305. The age distribution of the respondents was (18-52) years while the mean age was 31.1±5.69). The age group with the largest number of respondents was the age group (30 - 39) years, which were 158 (51.8%). The greater proportions of the respondent were male 158 (51.8%) while female accounted for 147(48.2%) giving a ratio of male to female of approximately 1.1:1. More than half of respondents were married 61.6% while the singles accounted for 36.4%. Respondents that were either widowed or divorced accounted for only 2% the study (Table 1). The majority of respondents were of the Yoruba tribe (89.2%) and Christians by religion (88.9%). Majority of the respondents had tertiary education (83.5%), 11.5% had secondary education and 2.6% had education up to primary school level. About 2.3% of the respondents has no form of education at all (Table 2). While majority (94.8%) of the respondents were aware of self-medication, only 47.2% of them showed good knowledge of self-medication. More than half (52.8%) of respondents demonstrated poor knowledge. Also, more than half (52.1%) of the respondents had practiced self-medication after self-diagnosis and about one third of them (31.8%) had practiced self -medication during three months prior to the study. Only 45.2% of respondents have ever repurchased and reused drugs without prescription, while 27.5% respondents have done this in the three months prior to this study. Only 34.1% of respondents have consulted a medical doctor in the 3 months before the study while 37.0% of respondents did so in the past twelve months. Almost a third of the respondents (28.9%) never consulted a doctor or got drugs on doctor’s prescription (Table 3). Drug types normally bought and used without prescription were: analgesics (38.2%), antibiotics (19.0%), anti-malarials (13.3%) and others (29.4%). Majority (53.4%) had no difficulty buying non prescribed drugs from the pharmacies and patent medicine outlets (Figure 1). The reasons for self-medication included: financial problems (10.8%), mild sickness (10.8%), lack of time (13.4%), knowledge of diagnosis (5.6%), convenience (2.3%), and non-availability of doctor (3.0%). Majority (63.0%) of the respondents had never had professional counselling against self- medication. Antimicrobial self-medication had been practiced in the preceding twelve months by 124(40.7%). Conditions for which antimicrobials were being used included; body pains (14.9%), catarrh (14.9%), headache (14.3%), sore throat (11.5%), diarrhea (11.2%), fever (9.0%), and toothache (5.6%).

**Table 1:** Socio demographic characteristics of respondents

Variable	Frequency	Percent
**Age Group**		
< 30 years	119	(39.0)
30 - 39 years	158	(51.8)
40 - 49 years	24	(7.9)
≥ 50 years	4	(1.3)
**Sex**		
Male	158	(51.8)
Female	147	(48.2)
**Marital Status**		
Married	188	(61.6)
Single	111	(36.4)
Widowed	4	(1.3)
Divorced	2	(0.7)
**Tribe**		
Yoruba	272	(89.2)
Hausa	13	(4.3)
Ibo	18	(5.9)
Others	2	(0.7)
**Religion**		
Christianity	271	(88.9)
Islam	23	(7.5)
Traditional	4	(1.3)
Others	7	(2.3)
**Education Complete**		
None	7	(2.3)
Primary	8	(2.6)
Secondary	35	(11.5)
Tertiary	255	(83.6)

**Table 2:** Cross tabulation of socio-demographic characteristics and knowledge of self-medication

	Knowledge Score	
	Poor (%)	Good (%)
**Sex**		
Male	87 (55.1)	71 (49.9)
Female	74 (50.3)	73 (49.7)
χ2=0.628, df=1, Pvalue = 0.409		
**Marital Status**		
Married	108 (57.4)	80 (42.6)
Single	49 (44.1)	62 (55.9)
Widowed	3 (75.0)	1 (25.0)
Divorced	1 (50.0)	1 (50.0)
χ2= 5.763, df=3, Pvalue = 0.124		
**Tribe**		
Yoruba	143 (52.2)	131 (47.8)
Hausa	10 (76.9)	3 (23.1)
Ibo	8 (44.4)	10 (55.6)
χ2=3.581, df=2, Pvalue = 0.167		
**Religion**		
Christianity	138 (50.9)	133 (49.1)
Islam	16 (69.6)	7 (30.4)
Traditional	7 (63.6)	4 (36.4)
χ2= 3.495, df=2, Pvalue = 0.174		
**Educational Level**		
None	6 (85.7)	1 (14.3)
Primary	8 (100)	0 (0)
Secondary	29 (82.9)	6 (17.1)
Tertiary	118 (46.3)	137 (53.7)
χ2=27.238, df=3, Pvalue = 0.000		
**Age**		
< 30 years	73 (61.3)	46 (38.7)
30 - 39 years	72 (45.6)	86 (54.4)
40 - 49 years	92 (55.1)	75 (44.9)
More than 50	4 (100)	0 (0)
χ2= 10.451, df=3, Pvalue = 0.015		

**Table 3:** Practice of Self-medication

Variable		Frequency	(Percent)
**Used any drug after self- diagnosis**			
	Yes	159	(52.1)
	No	146	(47.9)
**How many times in the Last 12 month**			
	None	188	(61.6)
	1 - 3	97	(31.8)
	4 - 6	17	(5.6)
	≥ 7	3	(1.0)
**Ever re-purchased and reused drugs without prescription**			
	Yes	138	(45.2)
	No	167	(54.8)
**How many times in the Last 12 month**			
	Not Applicable	205	(67.2)
	1 - 3	84	(27.5)
	4 - 6	10	(3.3)
	≥ 7	6	(2.0)
**Taking any drugs presently**			
	Yes	76	(24.9)
	No	229	(75.1)
**Drugs obtained after Doctors consultation and prescription**			
	Yes	122	(40.0)
	No	183	(60.0)
**Last time you consulted and got drugs on doctor’s prescription**			
	Within last 3month	104	(34.1)
	Within last 12 month	113	(37.0)
	Never	88	(28.9)
**Drug type normally bought and use without prescription**			
	Analgesic	187	(38.2)
	Anti malaria	65	(13.3)
	Antibiotics	93	(19.0)
	Others	144	(29.4)
**Difficulty buying non prescribed drugs**			
	Yes	41	(13.4)
	No	163	(53.4)
	No response	101	(33.1)

## Discussion

Self-medication is a component of self- care recognized by World Health Organization [2]. Positively, it helps patient to take care of minor ailment, reduces pressure on medical services especially in area where personnel are insufficient. If done correctly, it reduces both cost and time spent in accessing health care. Negatively, self-medication has been viewed as a human malpractice with increased risk of adverse drug reactions, drug interactions, inadequate dosing, polypharmacy, course and indiscriminate drug use [14]. All these contribute to the emergence and spread of antimicrobial drug resistance, this further leads to loss of cheap effective drugs and demand for newer expensive drugs [6]. The World Health Organization recommended doctor to patient ration is 1:1000 but in Nigeria it is 28 doctors to 100,000 patient [15]. This implies that the number of patients who have access to doctors are few and it further implies that the few doctors will be stretched and patient will most likely resort to self-medication. Another problem that is peculiar to Nigeria is the availability and uncontrolled access to all kinds of medicines especially prescription-only type. The findings of this study indicate that the prevalence of self-medication is still high (52.1%). This is similar to the study in Cameroon (55.7%) [16] and Pakistan (51%) [14]. These may be because they are developing countries with few healthcare facilities and personnel. Most of the available healthcare facilities are located in the urban centers while majority of the population still live in the rural area where healthcare facilities are grossly inadequate. The prevalence of self-medication in some countries like USA, Denmark, Spain and Lithuania is very low (17%, 3%, 11% and 22%) respectively [12]. These countries are well developed with advanced health care, adequate personnel compared with developing countries. Restricted legislated control of prescription-only drugs and over the counter drugs may account for this difference in prevalence. The prevalence of self-medication from several Nigerian studies ranges between 60-90%. Among undergraduate students of a Nigerian university, the prevalence of self-medication was 67% [17] and in Lagos, south-west Nigeria, self-medication was reported in 67.7% of infants being treated for colic [18]. Among patients attending the general outpatients and dental clinics in Owo, Nigeria self-medication was reported in 85% and 79% respectively [19, 20]. A study amongst health workers in tertiary hospital in Ondo State, Nigeria reported a prevalence of 73% [20]. The difference in prevalence in the cited Nigerian studies could be ascribed to the difference in sample size, sampling population, survey location and sampling method. Studies from previous studies reported prevalence rates ranging from 73.9% to 92% [7, 19]. This significant difference in prevalence rate when compared to this study could be due to the fact that most of these other studies were carried out among the general population while ours was done among healthcare workers who have more knowledge about health issues. In studies from Sudan and Nepal with high prevalence of self-medication, education status, socio-economic status, gender and age appears to be major factors associated with self-medication. Self-medication was higher with more advanced education, more in middle income earners, female and those below age of 40 [6, 21–23].

This is similar to our finding in this study for ages above 49yrs (P value =0.015). They practice self-medication more because they are active, richer than extremes of ages. In the contrast to the above findings, those with lower education among our cohort practice self-medication more (P value=0.000). These may be because of difference in sample population. It was observed that, in contrast to the Sudanese study that the male sex has significant association to self-medication. Unlike this study which only shows that males practice self-medication more than females but it is not significant. There was no significant association that was observed in other socio-demographics characteristic like marital status, tribe and religion. However majority of the respondents were Yoruba by tribe and Christian by religion. This is in keeping with the predominant tribe and religion in this region of Nigeria. Majority (94.8%) of the respondents were aware of self-medication but only 47.2% of respondents had good knowledge, hence there is need for an encompassing health education of healthcare workers and the general population at large. This finding is similar to that of several studies [1, 24, 25]. Majority (53.4%) had no difficulty in buying non-prescribed drug from various outlets and 63% of respondents were not counseled by health professional against self-medication (63.0%). The implication is that majority of the respondents practice self- medication and they are unguided. Health professionals should be mandated to educate their patients on the danger and problems associated with self-medication.

## Conclusion

The prevalence of self-medication is high among healthcare workers in Ido-Ekiti, South-West Nigeria. Efforts must be made to educate not just the health workers but the general populace on the disadvantages and complications of self-medication. There is also the need for legislation and enforcement of existing laws to discourage uncontrolled access to prescription only medications while over-the -counter (OTC) drugs should be used only when there is an absolute need.

### What is known about this topic

- A high prevalence of self medication has been documented across the globe;
- Inappropriate self-medication is a global problem that results in wastage of resources;
- Antimicrobial resistance is a pitfall of self medication.

### What this study adds

- The prevalence of self medication is still high even among health workers;
- Awareness is high (94.8%) but in-depth knowledge of self medication is low (47.2%);
- Easily accessible drugs such as antibiotics, antimalarial and analgesics are the most abused.
